# Tailoring of Mesoporous Silica-Based Materials for Enhanced Water Pollutants Removal

**DOI:** 10.3390/molecules28104038

**Published:** 2023-05-11

**Authors:** Daniela Flores, C. Marisa R. Almeida, Carlos R. Gomes, Salete S. Balula, Carlos M. Granadeiro

**Affiliations:** 1 LAQV-REQUIMTE, Departamento de Química e Bioquímica, Faculdade de Ciências, Universidade do Porto, Rua do Campo Alegre, s/n, 4169-007 Porto, Portugal; 2 Centro Interdisciplinar de Investigação Marinha e Ambiental (CIIMAR), Universidade do Porto, Terminal de Cruzeiros do Porto de Leixões, Av. General Norton de Matos s/n, 4450-208 Matosinhos, Portugal

**Keywords:** mesoporous silica, adsorption, water pollutants, toxic metal ions, organic dyes, environmental remediation

## Abstract

The adsorptive performance of mesoporous silica-based materials towards inorganic (metal ions) and organic (dyes) water pollutants was investigated. Mesoporous silica materials with different particle size, surface area and pore volume were prepared and tailored with different functional groups. These materials were then characterised by solid-state techniques, namely vibrational spectroscopy, elemental analysis, scanning electron microscopy and nitrogen adsorption–desorption isotherms, allowing the successful preparation and structural modifications of the materials to be confirmed. The influence of the physicochemical properties of the adsorbents towards the removal of metal ions (Ni^2+^, Cu^2+^ and Fe^3+^) and organic dyes (methylene blue and methyl green) from aqueous solutions was also investigated. The results reveal that the exceptionally high surface area and suitable ζ-potential of the nanosized mesoporous silica nanoparticles (MSNPs) seem to favour the adsorptive capacity of the material for both types of water pollutants. Kinetic studies were performed for the adsorption of organic dyes by MSNPs and large-pore mesoporous silica (LPMS), suggesting that the process follows a pseudo-second-order model. The recyclability along consecutive adsorption cycles and the stability of the adsorbents after use were also investigated, showing that the material can be reused. Current results show the potentialities of novel silica-based material as a suitable adsorbent to remove pollutants from aquatic matrices with an applicability to reduce water pollution.

## 1. Introduction

In recent decades, environmental pollution has been increasing exponentially, being a global problem that needs to be urgently mitigated [1]. The intensification of human activities, the increase in global population and the progress of modern industry are responsible for generating approximately 1.3 billion tons of solid waste annually with severe consequences to the environment [2]. Water is an indispensable natural resource for life on our planet and, therefore, providing clean and safe water through pollutant prevention or remediation processes is a vital global challenge for our society [3,4]. Water contaminants are typically divided into inorganic toxic elements, organic compounds and microorganisms [5]. Regarding inorganic contamination, toxic metal ions (Pb^2+^, Cu^2+^, Zn^2+^, Hg^2+^, Ni^2+^) are of particular concern due to their notorious toxicity and carcinogenicity, even at 1–100 µM levels and bioaccumulation in living organisms [6,7]. Organic water pollutants typically include pharmaceuticals, detergents, personal care products, pesticides and organic dyes, which are becoming increasingly more present in water effluents as a consequence of the industrial development and human activities as well as their environmental persistency [8]. For instance, organic dyes arising mainly from the textile, leather, wood, paper and pharmaceutical industries are heavily discarded into water effluents with harmful consequences for aquatic ecosystems and ultimately to human health [9]. In recent years, several water treatment technologies have been proposed for the efficient removal of pollutants from wastewater while avoiding secondary pollution [10,11]. Adsorption is considered one of the most promising techniques for water remediation due to its simple operation process, high efficiency, low cost, versatility and low consumption of energy [3,11,12]. A wide variety of adsorbents have been developed for the removal of water pollutants, such as activated carbons, zeolites, anionic clays, carbon nanomaterials, aluminium phosphates and metal–organic frameworks [3,5,13,14,15,16,17,18,19]. Nevertheless, some disadvantages associated with these materials have already been identified, such as low surface area, difficulty in the fine-tuning of their physicochemical properties, low adsorption capacity and reduced stability in water [20]. Thus, there is still a need to prepare new porous adsorbent materials with improved adsorption capacities and high structural robustness in aquatic media for cost-efficient environmental remediation systems.

Mesoporous silica has received enormous attention due to its structural and economic features, namely the fast adsorption of several water pollutants (metal ions, organic dyes, pesticides, pharmaceuticals), high surface area (200–1500 m^2^·g^−1^), uniform pore size distribution and exceptional chemical, thermal and mechanical stabilities [10,21,22,23]. Radi et al., developed a novel hybrid adsorbent based on mesoporous silica that functionalised with amino pentacarboxylic acid for the removal of toxic metal ions from water [10]. The adsorption studies revealed that the metal ion uptake by the mesoporous adsorbent is a highly efficient (sorption capacity of 2.40 mmol/g for Cu^2+^) and fast process, reaching equilibrium after only 20 min of contact time. Microporous silica molecules bearing small channels and pore sizes (<2 nm) have been extensively studied and used in ion exchange, adsorption, separation and industrial catalysis. However, this type of material is not able to meet the needs for applications in which macromolecules are involved, since they are limited by their reduced pore accessibility [24]. On the other hand, the simple synthetic modulation of mesoporous silica allows us to tune its structural architecture, morphology and properties, making them highly suitable candidates for the adsorptive uptake of water pollutants [25,26,27]. Several studies have emerged on the application of the different types of mesoporous silica on the removal of water pollutants. It is well reported in the literature that the functionalisation of mesoporous silica with specific functional groups allows the fine-tuning of the adsorptive properties of the materials [28]. The grafting of functional groups at the surface or inside the porous channels of mesoporous silica allows the adsorption efficiency to be enhanced while also promoting selectivity towards target adsorbates [29,30]. Lee et al., have investigated the influence of thiol- and amino-functionalisation in mesoporous SBA-15 with different morphologies on the adsorptive removal of metal ions (Cu^2+^, Pb^2+^) from water [31]. The results revealed that the amine-functionalised adsorbents exhibit a stronger affinity towards Cu^2+^, while thiol modification favours the adsorption of Pb^2+^. Bail and co-workers reported the solvothermal synthesis of spherical mesoporous silica and its application in the removal of cationic dyes [32]. The condensation of silanol to siloxane groups by a post-synthetic thermal treatment made the adsorbent surface less sensitive to pH, thus enabling the efficient adsorption of methylene blue (83.8% removal) even at extreme acidic conditions (pH < 1).

This work is intended to provide an in-depth survey of the main factors affecting the adsorptive performance of mesoporous silica towards inorganic and organic water pollutants. For that purpose, distinct mesoporous silica-based materials were prepared with different physicochemical features, namely particle size, surface area, pore size and functionalisation with distinct organic groups. Mesoporous silica nanoparticles (MSNPs) possessing high surface area and large-pore mesoporous silica (LPMS) with wide pore diameters were prepared and their surfaces were modified with (3-aminopropyl)triethoxysilane (APTES) and 3-(triethoxysilyl)propyl isocyanate (TESPIC) functional groups. These mesoporous silica-based materials were evaluated as adsorbents for the removal of inorganic (metal ions) and organic (dyes) water pollutants. Adsorption studies for the uptake of metal ions and organic dyes were conducted in order to evaluate the influence of physicochemical properties, namely surface charge and textural properties, on the adsorptive performance of the adsorbents. Furthermore, the adsorption kinetics, recyclability and stability of the adsorbents were also assessed.

## 2. Results and Discussion

### 2.1. Characterisation of the Materials

Different mesoporous silica-based materials were prepared with small particle size, mesoporous silica nanoparticles (MSNPs) and with enhanced porosity and large-pore mesoporous silica (LPMS). MSNPs were prepared using Pluronic F-127 as a non-ionic surfactant and triethanolamine as co-inhibitor of particle growth. On the other hand, LPMS were synthetised using Brij-56 as template and ethyl acetate as swelling agent with TEOS as the silica precursor. Moreover, both mesoporous materials were also functionalised using two different functional groups: 3-(aminopropyl)triethoxysilane (APTES) or 3-(triethoxysilyl)propyl isocyanate (TESPIC) as grafting agents. By doing so, we aimed to evaluate the influence of several parameters, namely particle size, surface area, pore diameter and the presence of functional groups on the adsorptive capacity of the materials towards metal ions.

The confirmation of the successful preparation of the mesoporous silica-based materials was achieved through vibrational spectroscopic analysis (FT-IR and FT-Raman), elemental analysis, scanning electron microscopy (SEM)/energy-dispersive X-ray spectroscopy (EDS) and textural analysis (nitrogen adsorption–desorption isotherms). 

The materials were initially characterised by FT-IR spectroscopy (Figure S1) and the assignment of the characteristic vibrational bands is summarised in Table S1. The FT-IR spectra of the prepared MSNPs and their corresponding functionalised materials are displayed in Figure S1A. All spectra display the intense characteristic bands of silica-type materials, in particular those associated with ν_as_(Si-O-Si), ν_s_(Si-O-Si) and δ(O-Si-O) vibrational modes located in the 1078–1074, 800–794 and 461–455 cm^−1^ ranges, respectively [22,33]. Moreover, the spectra also confirm the successful functionalisation of MSNPs by exhibiting the bands associated with the alkyl chains of the ammonium groups. In particular, the stretching modes of CH_2_ resulted in bands at 2929 cm^−1^ for APTES groups and at 2931 and 2980 cm^−1^ for TESPIC groups [34,35]. The peaks located at 1635 and 1558 cm^−1^ are assigned to the N-H bending mode of APTES groups and at 1574 cm^−1^ for TESPIC groups [36,37].

The FT-IR spectra of synthesised LPMS and its functionalised analogues are displayed in Figure S1B. Once again, the spectra are dominated by the intense silica bands associated with the ν_as_(Si-O-Si), ν_s_(Si-O-Si) and δ(O-Si-O) vibrational modes located in the 1105–1076, 808–805 and 471–456 cm^−1^ ranges, respectively [22,33]. The presence of additional bands in the spectra of functionalised materials points out to the successful functionalisation of LPMS. In fact, the bands associated with the δ(N-H) of functional groups can be observed at 1558 and at 1541 cm^−1^ for LPMS-APTES and LPMS-TESPIC, respectively. Additionally, the ν(C-H) stretching mode of the alkyl chains from the functional groups can be observed at 2958 and 2989 cm^−1^ for LPMS-APTES and LPMS-TESPIC, respectively [37]. 

**Figure 1:**
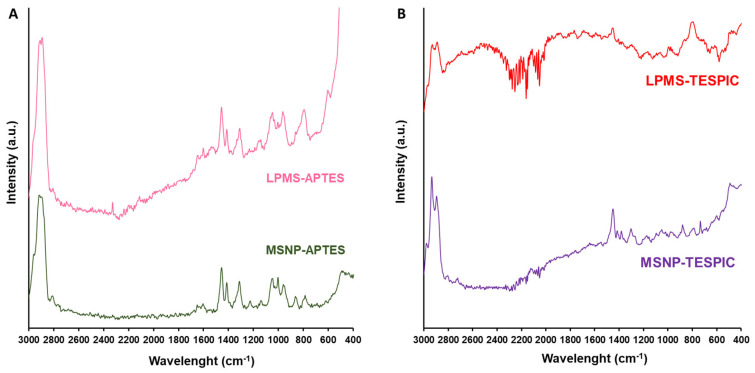
FT-Raman spectra of MSNP and LPMS materials functionalised with (**A**) APTES and (**B**) TESPIC groups. See the Experimental Section for materials’ abbreviation meanings.

FT-Raman spectroscopy is an extremely useful technique for the characterisation of functionalised silica-based materials, owing to the weak Raman signal provided by the siliceous bands. For that reason, the FT-Raman spectra of the functionalised materials (Figure 1) are mainly composed of the bands arising from the alkyl fragments of the functional groups (APTES and TESPIC). For the APTES-functionalised materials (Figure 1A), these include the bands in the 2800–2950 cm^−1^ region that are assigned to the ν(C–H) stretch, as well as the bands at 1604 cm^−1^, 1415 cm^−1^, 1313 cm^−1^ and 1003 cm^−1^, which are associated with δ(NH_2_), δ(CH_2_)_twist_, ν(CH_2_) and ν(C–C) modes, respectively [38]. Regarding the FT-Raman spectra of the TESPIC-modified materials (Figure 1B), the bands arising from the ν(C–H) stretch of the functional group are exhibited in the 2700–3100 cm^−1^ range. Furthermore, the spectra exhibit the bands associated with the ν_s_(C=O), δ(CH_2_), ν(C–C) and ν(Si –O–Si) vibrational modes at 1446, 1413, 1299 and 788 cm^−1^ for LPMS-TESPIC and at 1450, 1416, 1302 and 785 cm^−1^ for MSNPs-TESPIC, respectively [39].

The successful functionalisation of the mesoporous silica-based materials was further confirmed by elemental analysis. Table 1 summarises the elemental analysis results for the functionalised silica materials. The results confirm the functionalisation of the mesoporous silica materials with each of the functional groups. Moreover, the results also indicate that for both types of mesoporous silica, a more efficient functionalisation could be achieved using APTES rather than TESPIC.

**Figure 2:**
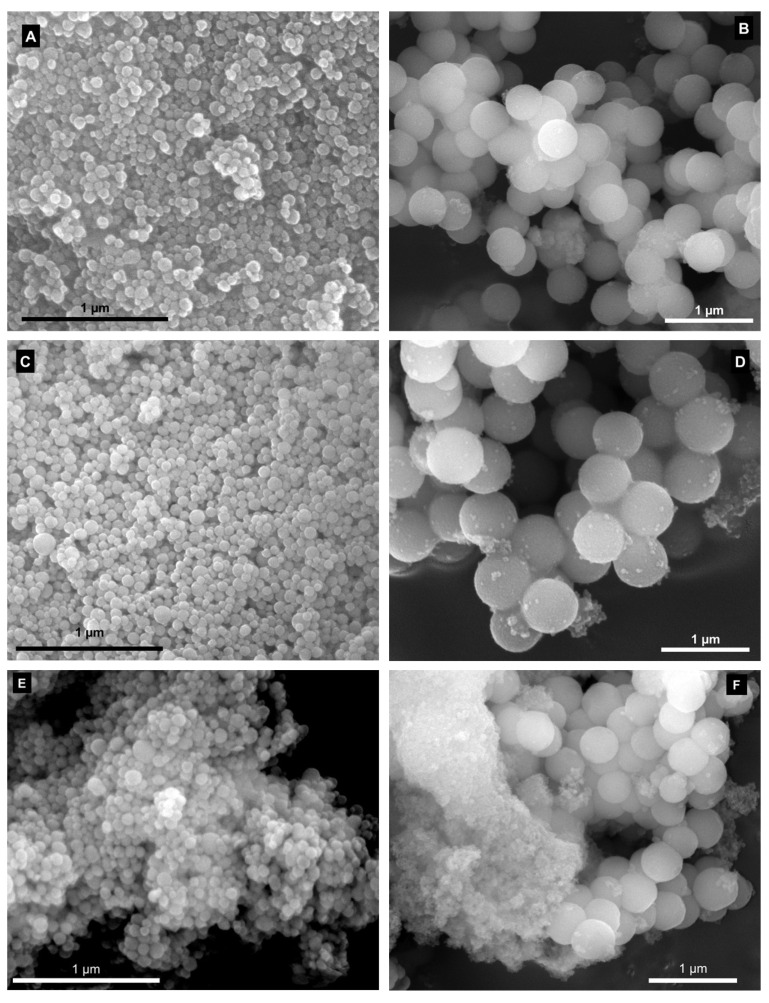
SEM micrographs of (**A**) MSNPs, (**B**) LPMS, (**C**) MSNPs-APTES, (**D**) LPMS-APTES, (**E**) MSNPs-TESPIC and (**F**) LPMS-TESPIC materials. See the Experimental Section for materials’ abbreviation meanings.

The morphology and chemical composition of the materials prior and after functionalisation were assessed by SEM/EDS techniques. As expected, SEM micrographs of MSNPs (Figure 2A) exhibit well-defined spherical nanoparticles with uniform size distribution and an average diameter of 65 ± 14 nm [22,40]. The SEM images of LPMS (Figure 2B) show that the sample is composed by evenly distributed spherical particles with an average diameter of 465 ± 78 nm, which is in good agreement with the literature [24,33]. The results also clearly indicate that the morphology and size distribution of the starting mesoporous silica have been preserved in all functionalised materials. In fact, MSNPs-APTES and MSNPs-TESPIC (Figure 2C,E) still exhibit the same type of nanospheres of the starting MSNPs with average diameters of 69 ± 18 and 78 ± 14 nm, respectively. Similarly, the SEM micrographs of LPMS-APTES and LPMS-TESPIC (Figure 2D,F) also display identical spherical particles to the initial LPMS with average diameters of 470 ± 60 and 452 ± 35 nm, respectively.

The obtained EDS spectra (Figure S2) reveal that the expected chemical composition for the silica materials display silicon as the main element, along with carbon and oxygen, as well as confirming the absence of any sample contamination.

The textural properties of porous materials are known to have a strong influence on their adsorptive capacity. For that purpose, nitrogen adsorption–desorption studies were performed for all the mesoporous silica-based materials. As expected, the nitrogen adsorption isotherms for all materials are of type IV classification with a sharp capillary condensation step in the relative pressure ranges of 0.90–0.99 and 0.80–0.99 for MSNP- and LPMS-type materials, respectively (Figure S3) [24,40]. The obtained textural parameters for the starting and functionalised materials are summarised in Table 2. The MSNP sample has shown a high BET surface area (S_BET_) of 913 m^2^/g, a pore volume (V_p_) of 1.64 cm^3^/g and a pore diameter (D_p_) of 5.95 nm. LPMS exhibited smaller BET surface area (138 m^2^/g) and pore volume (0.75 cm^3^/g), although revealing a remarkable pore diameter of 16.36 nm that confirms the presence of ultra-large pores in the material. The addition of organic functionalities has led to the simultaneous decrease in S_BET_ and V_p_ when compared with the parent materials, thus confirming their successful functionalisation and the presence of the functional groups inside the porous channels of mesoporous silica. In summary, the textural properties are in good agreement with the literature, reflecting the considerably higher surface area-to-volume ratio of nanosized MSNP materials as well as the ultra-large pores in LPMS materials [24,40].

The colloidal stability of the mesoporous silica-based materials was investigated by studying the pH-dependence of the surface charge and hydrodynamic size. It is well known that the electrostatic interaction between charged silica particles and metal ions is the main driving force for the adsorption process [41]. For that reason, the influence of pH on the surface charge of the materials was studied through the measurement of their zeta (ζ) potential (Figure S4). As expected, the modification with organic groups (APTES and TESPIC) has led to an increase in ζ-potential when compared with the parent materials (MSNPs and LPMS) [42,43]. The results show that, among the functionalised materials, the surface modification with APTES resulted in the most significant increase in zeta potential. It can be observed that, for all materials, the zeta potential shifts from positive to negative values with increasing pH [44]. The pH_PZC_ value (pH at the point of zero charge) was also determined for the studied materials. A pH_PZC_ value of 2.2 was calculated for MSNPs, meaning that for pH > pH_PZC_, its surface will be negatively charged, hence more available to adsorb cationic metal ions by electrostatic interaction. The introduction of TESPIC groups has led to a pH_PZC_ increase to 3.5 while functionalisation with APTES resulted in a pH_PZC_ of 8.0. A similar trend could be observed for the LPMS-type materials, exhibiting pH_PZC_ values of 2.1, 4.9 and 9.2 for LPMS, LPMS-TESPIC and LPMS-APTES, respectively (Table 3).

The hydrodynamic size of the materials was determined by dynamic light scattering (DLS) over a 3–13 pH range (Figure S5). The results obtained for the hydrodynamic size match well the previously observed variation of zeta potential along the pH range. In fact, for pH values close to pH_PZC_, a significant increase in the hydrodynamic size was observed for all materials. At such pH values, the extremely low surface charge leads to an agglomeration of the particles, thus reducing their colloidal stability. MSNPs and MSNPs-TESPIC suspensions were stable for pH values higher than 4 and 6, respectively, showing negligible agglomeration. The DLS results of MSNPs-APTES reveal good colloidal stability, although, as expected, the formation of large particle agglomerates was detected in the pH range close to pH_PZC_ (8–10). On the other hand, LPMS-based materials exhibited a good colloidal stability for pH > 6 without significant agglomeration by displaying hydrodynamic sizes close to the average diameter of the particles (determined by SEM).

### 2.2. Metal Ions Adsorption Studies

**Figure 3:**
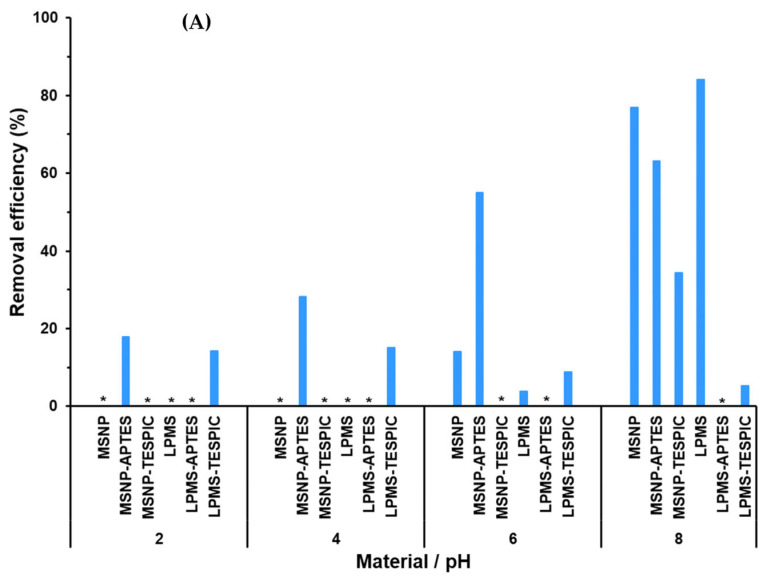

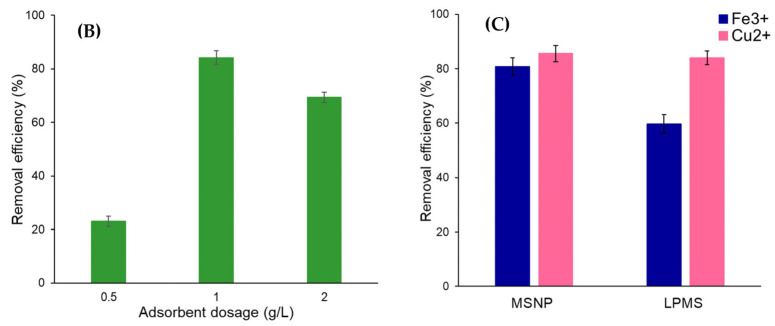
(**A**) Influence of pH on the removal of Ni^2+^ (1 mg L^−1^) from aqueous solution by the different mesoporous silica materials (1 g/L), (**B**) influence of LPMS adsorbent dosage on the removal of Ni^2+^ (1 mg L^−1^) at pH = 8 and (**C**) removal efficiency for Fe^3+^ and Cu^2+^ (1 mg L^−1^) by MSNPs and LPMS (1 g/L) at pH = 10. All experiments were conducted with a contact time of 2 h at room temperature. Mean and standard (*n* = 3) are shown. * denotes values below the detection limit (0.28 mg L^−1^). See Experimental Section for materials’ abbreviation meanings.

The mesoporous silica-based materials were evaluated as adsorbents for the removal of some of the most commonly found metal ions (Ni^2+^, Fe^3+^, Cu^2+^) in polluted water. Metal ions in water are typically stable at low pH levels, although with increasing pH, they tend to react with hydroxide ions and precipitate as metal hydroxides [45]. To avoid interference from metal hydroxide precipitation during the adsorption process, it is crucial to determine the pH range at which chemical precipitation occurs. For that reason, blank experiments were conducted prior to the application of the materials in the adsorption of metal ions. Blank tests were performed in the absence of the adsorbents to investigate the occurrence and extent of chemical precipitation over a 2–12 pH range for the selected metal ions. The results obtained for Ni^2+^ reveal the occurrence of extensive chemical precipitation (>92%) for pH ≥ 10 (Figure S6). The increasing amount of hydroxide ions at alkaline pH leads to the chemical precipitation of Ni^2+^ in the form of Ni(OH)_2_ [46]. Nevertheless, very low or even no precipitation could be detected over the 2–8 pH range, making it suitable for the adsorption studies of this metal ion. For that reason, we have initially performed an extensive survey by testing all the mesoporous silica-based adsorbents in the adsorption of Ni^2+^ over the selected pH range (Figure 3A).

The results indicate that all the prepared materials, with the exception of LPMS-APTES, showed some adsorptive capacity towards Ni^2+^ along the studied pH range. As previously discussed, LPMS-APTES exhibited positive ζ-potential for pH ≤ 8, which should be the main reason for the absence of adsorption as a result of repulsive electrostatic interactions with the positively charged metal ion. Negligible or undetected adsorption was observed at highly acidic media, probably because the high amount of H^+^ ions compete with metal ions for the active spaces of the adsorbent [47]. In general, it seems that the more favourable conditions for the removal of Ni^2+^ occur at pH = 8. The best adsorptive performances could be attained with LPMS and MSNPs as adsorbents with removal efficiencies of 84 ± 1 and 77 ± 1%, respectively. Despite exhibiting similar ζ-potential at pH = 8 (Figure S4), MSNPs showed higher S_BET_ and V_p_ than LPMS (Table 2); therefore, it would have been expected to present a superior adsorptive capacity. Nevertheless, the considerably higher D_p_ value of LPMS (16.36 nm) seems to play a crucial role in Ni^2+^ removal since the wider pores of LPMS are expected to allow an easier diffusion of metal ions into the porous framework while avoiding pore blockage issues.

The adsorbent dosage is a crucial factor for the removal efficiency, and the most suitable dosage for the adsorption system should be determined. The effect of adsorbent dosage in the removal of Ni^2+^ was investigated using LPMS as adsorbent, varying its mass while keeping other experimental conditions unchanged (pH = 8, metal ion concentration 1 mg L^−1^, contact time of 2 h). As expected, the adsorption results reveal that, with the increase in adsorbent dosage from 0.5 to 1.0 g/L, the removal increases from 23 ± 2% to 84 ± 1% most likely due to the increase in surface area and vacant sites (Figure 3B) [48]. However, a further increase in the adsorbent dosage from 1.0 to 2.0 g/L did not enhance Ni^2+^ removal. Previous studies have reported that the continuous increase in adsorbent dosage ultimately leads to a reduction in metal ion uptake due to the aggregation of active sites [49,50]. The obtained results indicate that 1.0 g/L provides enough surface area and available sites for the adsorption of the metal ion and was considered as the suitable adsorbent dosage.

Additional adsorption studies for the removal of other metal ions (Fe^3+^, Cu^2+^) were conducted by using the adsorbents which exhibited the best removal efficiencies for Ni^2+^. The experimental conditions have been determined by considering both the ζ-potential of the adsorbents (Figure S4) and the occurrence of chemical precipitation (Figure S6). The more acidic conditions (pH = 2) were excluded based on the positive ζ-potential of both adsorbents as well as the 4–8 pH range based on the extensive chemical precipitation detected for Fe^3+^ and Cu^2+^. Between the remaining pH values, the lower value (pH 10) was selected to avoid a possible degradation of the mesoporous materials in highly alkaline conditions. The results show that both adsorbents also exhibit a strong affinity towards these metal ions, being able to efficiently remove them from aqueous media within a considerably short period of time (Figure 3C). In particular, MSNPs were able to reach efficiencies of 81 ± 2% and 86 ± 3% for the removal of Fe^3+^ and Cu^2+^, respectively. Despite the expected chemical precipitation of Cu^2+^ at the selected pH (see Figure S6C), both adsorbents were still able to further remove a significant amount of metal ion, making the proposed mesoporous adsorbents very promising in the development of adsorption systems that are able to meet the strict current water quality standards for the selected metal ions.

### 2.3. Dye Adsorption Studies

**Figure 4:**
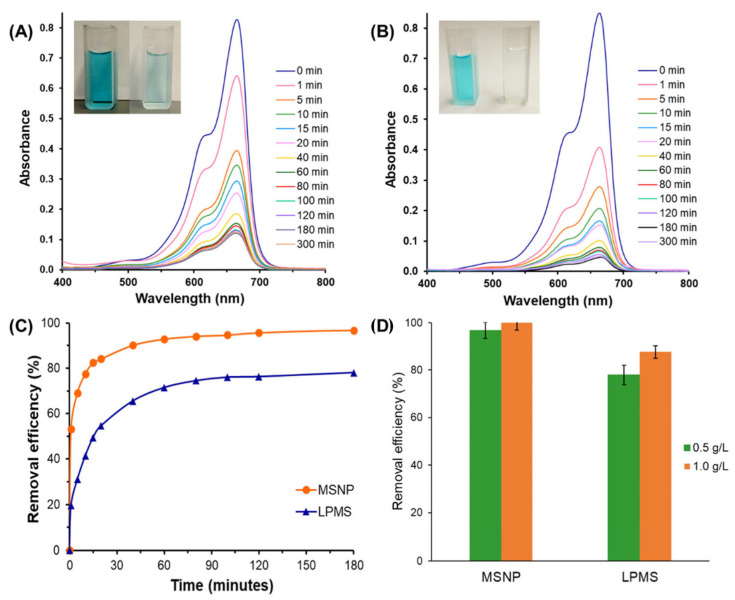
Evolution of UV–Vis absorption spectra for methylene blue (MB) as a function of time using (**A**) LPMS and (**B**) MSNPs as adsorbents (insert shows the dye solution before and after adsorption); (**C**) time-dependent adsorption performance for MB (5 mg L^−1^) by MSNPs and LPMS (0.5 g/L) and (**D**) influence of adsorbent dosage of MSNPs and LPMS on the removal of MB (5 mg L^−1^) with a contact time of 2 h (10 min for MSNPs 1.0 g/L) at room temperature. Mean and standard (*n* = 3) are shown. See the Experimental Section for materials’ abbreviation meanings.

The adsorption capability of the prepared adsorbents to remove organic pollutants from water was evaluated for two organic dyes with different sizes and functional groups, methylene blue (MB) and methyl green (MG). The adsorption studies were carried out by monitoring the characteristic absorption band of each dye in the UV–Vis spectra (663 nm for MB and 635 nm for MG). The decrease in absorbance intensity over time indicates the decrease in dye amount in solution, which was determined using calibration curves obtained with aqueous standard solutions of each dye. The adsorptive performance of all mesoporous silica materials was investigated for the removal of MB (5 mg L^−1^) using a fixed adsorbent dosage (0.5 g/L) and pH (8). The evolution of the UV–Vis spectra using LPMS and MSNPs as adsorbents (Figure 4A,B) clearly shows a fast MB removal by both materials associated with a naked-eye detectable loss of colour. Among the studied materials, MSNPs showed a higher adsorptive capacity, being capable of removing 97 ± 2% of the dye from solution after 2 h (Figure S7). Despite exhibiting smaller S_BET_, the presence of ultra-large pores in LPMS also allowed a removal efficiency of 78 ± 2% to be reached for the same contact time. Overall, the introduction of positively charged APTES groups on mesoporous silica drastically hinders the adsorption of cationic MB molecules as a result of strong repulsions between the dye and silica surface, which is in good agreement with the ζ-potential results [51]. The TESPIC-functionalised mesoporous silicas exhibited a less efficient adsorptive capacity when compared with the parent materials, which can be attributed to their less negative ζ-potential at the chosen pH together with the reduced S_BET_ and V_p_ after functionalisation.

The MB removal by MSNP and LPMS materials (Figure 4C) increased rapidly in the initial 20 min due to the complete availability of the pores, followed by a more gradual increase, until reaching equilibrium at 2 h. The initial adsorbent dosage plays an important role in the adsorptive performance of a material towards organic dyes. For that reason, the influence of the adsorbent dosage of MSNPs and LPMS on their adsorptive performance was evaluated by maintaining the other experimental parameters unchanged. The results show that increasing the adsorbent dosage from 0.5 to 1.0 g/L leads to a significant enhancement of MB removal for both adsorbents (Figure 4D). In fact, the efficiency of LPMS increased from 78 ± 2% to 88 ± 3% of MB removal after 2 h, while the time needed to reach complete MB removal using MSNPs could be reduced from 2 h to only 10 min of contact time.

#### 2.3.1. Adsorption Kinetics

The adsorption kinetic provides valuable information on the adsorption mechanism, allowing us to accurately determine the adsorption rate during pollutant removal. The results reveal different adsorptive capacities according to the type of adsorbent and dye (Figure S8). The nanosized MSNPs exhibited a higher adsorptive capacity for both dyes, reaching adsorption equilibrium within only 20 min, while the LPMS-based system requires 2 h for equilibrium to be reached. The obtained data were fitted with the linear forms of the pseudo-first-order (Equation (3)) and the pseudo-second-order (Equation (4)) models [52,53,54,55]. The fitting results are displayed in Figure S9 and the calculated kinetic parameters are summarised in Table 4. For all the kinetic studies, the correlation coefficients (R^2^) obtained by the pseudo-first-order model (0.651–0.985) are considerably lower than the ones obtained through the pseudo-second-order model (≥0.992). Moreover, the calculated q_e_ values by the pseudo-second-order model are also very close to the q_e_ values experimentally obtained. The results indicate that the adsorption of the organic dyes (MB and MG) by the mesoporous silica adsorbents is better described by the pseudo-second-order model, suggesting that the rate of the adsorption process is controlled by the chemisorption process [56,57].

#### 2.3.2. Recyclability and Stability of the Adsorbents

**Figure 5:**
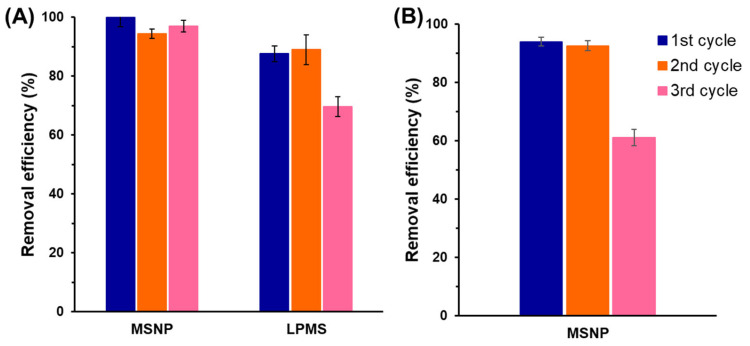
Recycling ability of the mesoporous silica adsorbents for the removal of (**A**) methylene blue (MB) and (**B**) methyl green (MG) with an initial concentration of 5 mg L^−1^, adsorbent dosage 1.0 g/L, contact time of 20 min and 2 h for MSNP and LPMS materials, respectively. Mean and standard (*n* = 3) are shown. See the Experimental Section for materials’ abbreviation meanings.

For practical applications, it is highly desirable to develop a cost-efficient adsorbent that is directly related to its regeneration ability [58,59]. The feasibility of the MSNP and LPMS adsorbents was evaluated by conducting recycling experiments in consecutive adsorption–desorption cycles for MB. Based on the previous extensive analysis, regeneration studies were conducted with an initial concentration of 5 mg L^−1^, an adsorbent dosage of 1.0 g/L, a pH of 8 and a contact time of 2 h. After each adsorption cycle, the adsorbents were separated, washed thoroughly with ethanol, dried and reused in a new cycle. The results show a remarkable recycling ability of MSNPs, being able to maintain a removal efficiency >94% in three consecutive cycles after only 30 min of contact (Figure 5). On the other hand, LPMS were able to maintain its adsorptive capacity in the second cycle, although a reduction of 70% was detected in the third cycle. The reduction in adsorptive capacity should be related to the occurrence of pore blockage as a result of the significantly smaller S_BET_ of LPMS when compared with MSNPs.

The superior adsorptive performance of MSNPs motivated their application in the recycling studies of the MG dye molecule under the previously established conditions. Once again, MSNPs exhibited a remarkable dye uptake by reaching removal efficiencies >92% after only 40 min up to the second adsorption cycle. In the third cycle, the efficiency was significantly reduced to 62 ± 2% most likely due to the bulkier dimensions of the MG molecule, which ultimately lead to the saturation of the mesoporous framework.

The stability of the material exhibiting better adsorptive performance (MSNPs) was assessed by performing the characterisation of the adsorbent after the adsorption studies. The FT-IR spectrum of the used adsorbent displays a very similar profile with the as-prepared material (Figure S10), while the SEM micrographs reveal that the morphology and size of the nanoparticles remain unchanged after the first or even the third adsorption cycle for MB and MG (Figure S11). The results confirm the exceptional robustness of MSNPs as an adsorbent under the experimental conditions without any evidence of degradation after adsorption studies.

Considering the adsorption results, together with previously reported studies, a possible adsorption mechanism can be suggested, involving chemical interactions [60]. Initially, cationic dyes move towards the external surface of the adsorbents in which boundary layer diffusion occurs. Afterwards, dye molecules diffuse into the internal mesoporous of silica-based adsorbents (internal diffusion). At this point, hydrogen bonding takes place between the hydroxyl groups (proton donors) of silica and the nitrogen atoms (proton acceptors) of cationic dyes [61,62,63]. Furthermore, electrostatic interactions may also occur during the adsorption process between the cationic dyes and the negatively charged surface of mesoporous silica adsorbents [64].

The performance of the adsorbents towards the adsorption of the studied organic dyes (MB and MG) was compared with other reported silica-based adsorbents (Table 5). Compared with the reported adsorbents that typically require one or more hours to reach equilibrium, the proposed MSNP adsorbent exhibits satisfactory adsorption capacities for both cationic dyes within a very short period of time. The reduced equilibrium time, together with the recyclability and stability of MSNPs, reveals their high potential for practical in-field applications.

## 3. Experimental Section

### 3.1. Materials and Methods

All the reagents used in the materials synthesis and functionalisation, such as Pluronic F-127 BioReagent (MW = 12,600 g/mol, Aldrich, St. Louis, MO, USA), n-cetyltrimethylammonium bromide ≥98% (CTAB, BDH Chemicals, Kuwait, Oman), triethanolamine 98% (TEA, Alfa Aesar, Haverhill, MA, USA), tetraethyl orthosilicate 98% (TEOS, Aldrich), polyoxyethylene 10 cetyl ether (Brij-56, MW ~683 g/mol, Aldrich), (3-aminopropyl)triethoxysilane ≥98% (APTES, Aldrich), 3-(triethoxysilyl)propyl isocyanate 95% (TESPIC, Aldrich), ethanol absolute 99.8% (EtOH, Fisher Chemical, Hampton, NH, USA), ethyl acetate (EA, Carlo-Erba, Cornaredo, Italy) and anhydrous toluene 99.8% (Aldrich) were purchased from chemical suppliers and used without further purification. The adsorption studies were performed using double-deionised water, hydrochloric acid ~37% (HCl, Fisher, Hampton, NH, USA), sodium hydroxide ≥98% (Aldrich) and metal standard solutions for AAS, namely copper (Panreac), nickel (Fluka) and iron (Aldrich). The dye adsorption studies were carried out with methylene blue hydrate ≥95% (MB, Riedel-de Haën, Seelze, Germany) and methyl green (MG, Aldrich).

For the characterisation of the adsorbent materials, Fourier-transform infrared (FT-IR) spectra were recorded in the 400–4000 cm^−1^ region on a Jasco FT/IR-460 Plus using KBr pellets while FT-Raman spectra were recorded on a RFS-100 Bruker FT-spectrometer equipped with a Nd:YAG laser with an excitation wavelength of 1064 nm and the laser power set to 350 mW. Scanning electron microscopy (SEM) and energy dispersive X-ray spectroscopy (EDS) studies were performed at the “Centro de Materiais da Universidade do Porto” (CEMUP, Porto, Portugal) using a Schottky, FEI Quanta 400 FEG ESEM scanning electron microscope equipped with an EDAX Genesis X4M energy-dispersive X-ray spectrometer. The adsorbent materials were studied as powders and were previously subjected to Au/Pd sputtering. The average diameter of the nanoparticles was determined by analysis of SEM images using ImageJ v.151 software (https://imagej.nih.gov/ij/; accessed on 5 February 2022; 50 measurements for each case). The zeta (ζ) potential of the particles were performed at 25 °C using a Malvern Zetasizer Nano-ZS scattering spectrometer, while the data were processed with the Malvern Dispersion Technology software 5.0 (Malvern Panalytical, UK). Textural properties were obtained from the physical adsorption of nitrogen at −196 °C using a Micromeritics ASAP 2010 instrument. For that, samples were degassed at 110 °C for 6 h prior to the measurements. The Brauner–Emmet–Teller (BET) surface area was determined using the relative pressure data in the 0.06–0.3 range. Total pore volume was calculated based on the amount adsorbed at the relative pressure of 0.99. CHN elemental analysis was performed on a Leco CHNS-932 instrument at the University of Santiago de Compostela.

In metal adsorption studies, the quantification of metals in solution was performed through atomic absorption spectroscopy in a Perkin Elmer (Norwalk, CT, USA) AAnalyst 200 spectrometer using calibration curves obtained from dilution of stock metal standard solutions with double-deionised water.

For organic dye adsorption studies, the organic dye concentration in solution was determined by UV–Vis using a Varian Cary 50 spectrophotometer by the calibration curve method.

### 3.2. Preparation of the Materials

#### 3.2.1. Mesoporous Silica Nanoparticles (MSNPs)

The mesoporous silica nanoparticles were prepared following the method described by Bouchoucha et al. [40]. Briefly, a mixture containing Pluronic F127 (2.68 g), CTAB (0.66 g) and TEA (15.64 g) was dissolved in EtOH (57 mL) and water (125 mL), and stirred for 24 h at room temperature. Afterwards, TEOS (2.56 mL) was added under vigorous stirring, and subsequently aged under static conditions for 24 h. Then, EtOH (100 mL) was added to promote precipitation. The solid was collected by centrifugation, dried at 80 °C and the resulting powder calcined in air at 550 °C for 6 h.

#### 3.2.2. Large-Pore Mesoporous Silica (LPMS)

The large-pore mesoporous silica was prepared following the method described by Chen et al. [24]. Initially, Brij-56 (4.83 g) was dissolved in 180 g of distilled water. Then, APTES (0.30 g) and EA (0.46 g) were added under stirring at room temperature. After stirring for 30 min, 4.80 g of TEOS was added and subsequently the mixture was stirred for 24 h at room temperature. Afterwards, the mixture was transferred to a closed container and heated for 24 h at 100 °C under static conditions. The resulting nanoparticles were collected by centrifugation and dried at 60 °C under vacuum for 48 h. The dried nanoparticles were calcined in air at 550 °C for 6 h.

#### 3.2.3. Functionalisation of Mesoporous Silica

The functionalisation of mesoporous silica (MSNPs and LPMS) was achieved following a previously reported procedure [22]. Initially, the mesoporous silica materials were dried at 120 °C under vacuum for 2 h in order to remove any physiosorbed water. The activated silica (250 mg) was refluxed with APTES or TESPIC (5 mmol) in anhydrous toluene (15 mL) for 24 h under inert atmosphere. The functionalised materials were isolated from the suspension by centrifugation, washed with anhydrous toluene and dried at 80 °C under vacuum for 2 h.

*MSNPs:* selected FT-IR (cm^−1^): 3403 (w), 1643 (w), 1078 (s), 969 (w), 794 (m), 456 (s).

*MSNPs-APTES:* Anal. Found (%): C, 10.72; N, 3.41; H, 2.81; loading of APTES: 2.43 mmol g^−1^ (12%); selected FT-IR (cm^−1^): 3419 (m), 2929 (m), 1635 (m), 1558 (m), 1491 (w), 1385 (s), 1354 (m), 1074 (vs), 795 (m), 696 (w), 573 (w), 461 (m); selected FT-Raman (cm^−1^): 2916 (s), 2899 (s), 2812 (w), 1651 (w), 1605 (w), 1456 (m), 1416 (m), 1313 (m), 1252 (w), 1140 (w), 1049 (m), 1003 (m), 961 (m), 864 (w), 795 (w), 492 (m).

*MSNPs-TESPIC:* Anal. Found (%): C, 9.85; N, 1.52; H, 2.14; loading of TESPIC: 1.08 mmol g^−1^ (5%); selected FT-IR (cm^−1^): 3408 (s), 2980 (m), 2931 (m), 2341 (w), 1635 (m), 1574 (m), 1456 (w), 1074 (s), 955 (m), 800 (m), 555 (w), 455 (s); selected FT-Raman (cm^−1^): 2980 (m), 2935 (s), 2897 (s), 2804 (w), 2725 (w), 1512 (w), 1450 (m), 1392 (m), 1302 (m), 1175 (w), 1051 (m), 1020 (w), 970 (m), 880 (m), 787 (w), 735 (m), 475 (m).

*LPMS:* selected FT-IR (cm^−1^): 2986 (w), 2164 (w), 1076 (s), 805 (m), 456 (s).

*LPMS-APTES:* Anal. Found (%): C, 6.10; N, 1.90; H, 1.57; loading of APTES: 1.35 mmol g^−1^ (7%); selected FT-IR (cm^−1^): 3448 (m), 2958 (m), 1871 (w), 1635 (w), 1558 (w), 1385 (s), 1105 (vs), 808 (m), 694 (w), 471 (s); selected FT-Raman (cm^−1^): 2910 (s), 2895 (s), 2808 (w), 1650 (w), 1603 (w), 1456 (m), 1414 (m), 1312 (m), 1242 (w), 1144 (w), 1047 (m), 1005 (m), 964 (m), 835 (w), 795 (w), 490 (m).

*LPMS-TESPIC:* Anal. Found (%): C, 4.53; N, 1.00; H, 0.99; loading of TESPIC: 0.72 mmol g^−1^ (4%); selected FT-IR (cm^−1^): 3446 (m), 2989 (w), 2941 (w), 1635 (m), 1541 (w), 1103 (s), 808 (w), 471 (m); selected FT-Raman (cm^−1^): 2970 (m), 2931 (s), 2891 (s), 2800 (w), 2729 (w), 1512 (w), 1452 (m), 1391 (w), 1298 (w), 1178 (w), 1051 (m), 1020 (w), 978 (m), 862 (m), 795 (s), 735 (w), 476 (m).

### 3.3. Adsorption Studies

Adsorption studies were carried out in glass vials with double-deionised water under controlled magnetic stirring at room temperature. For metal adsorption studies, different concentrations of each metal were added to the vials. After a specific contact time, aqueous solutions were collected for metal analysis. Organic dye-adsorption studies were carried out in glass vials with double-deionised water under dark conditions and controlled magnetic stirring at room temperature after adding the dye solution into the vials. Each test was replicated three times. At the end of all experiments, the adsorbent material was removed by centrifugation and the aqueous solutions were stored at 4 °C with 1% (*v*/*v*) of HNO_3_ for metal analysis. For the organic dyes, aqueous solutions were stored protected from light at room temperature.

The removal efficiency and adsorbed amount (*q_t_*) of each compound, metal or organic dye from solution after contact with the adsorbent were calculated as follows:

(1) $$Removal\;efficiency\;\left(\%\right)=\frac{\left(C_{0}-C_{t}\right)}{C_{0}}\times 100$$

(2) $$q_{t}\left({\mathrm{mgg}}^{-1}\right)=\frac{\left(C_{0}-C_{t}\right)}{m}V$$

where *C*_0_ and *C_t_* (mg g^−1^) are the initial concentration and the concentration at time *t*, respectively, of the compound; *V* is the volume of the solution (L) and m is the weight of the adsorbent (g). 

For the metal ions adsorption studies, blank experiments were performed using aqueous standard solutions (1 mg L^−1^) of each metal ion at different pH (2–12) for 5 h.

The effect of pH on metal adsorption (1 mg L^−1^) was investigated using a fixed adsorbent dosage (1 g/L) for a contact time of 2 h with pH monitoring and small additions of HCl or NaOH solutions were made whenever necessary. For metal ions, studies on the effect of the adsorbent dosage in solution (0.5–2.0 g/L) were carried out at fixed pH (8) and metal ion concentration (1 mg L^−1^). Studies on the effect of the adsorbent dosage (0.5–1.0 g/L) on organic dye adsorption were also performed at a fixed organic dye concentration (5 mg L^−1^).

The adsorption kinetics was evaluated to predict the organic dye removal rate and provide information about the mechanisms that control the adsorption process, being modelled using pseudo-first-order (PFO) and pseudo-second-order (PSO) rate laws. The linear forms of PFO and PSO can be represented as follows, respectively:

(3) $$\ln\left(q_{e}-q_{t}\right)=\ln\left(q_{e}\right)-k_{1}t$$

(4) $$\frac{t}{q_{t}}=\frac{t}{q_{e}}+\frac{1}{k_{2}q_{e}^{2}}$$

where *q_t_* and *q_e_* are the amount of adsorbed dye at any given time, *t*, and at equilibrium (mg g^−1^), respectively. *k*_1_ (min^−1^) and *k*_2_ (g mg^−1^ min^−1^) are the pseudo-first-order and pseudo-second-order rate constants, respectively [52,53,54,55].

To evaluate the possible recyclability of the adsorbents towards organic dyes, the adsorbent recovered by centrifugation was washed with acetonitrile, dried under vacuum and reused in a new adsorption experiment under identical experimental conditions.

## 4. Conclusions

The present work describes the tailoring of mesoporous silica-based materials by varying different parameters, namely particle size, surface area, pore size, pore diameter and surface functionalisation, evaluating, afterwards, their influence on the adsorptive capacity of the materials towards water pollutants. Nanosized and large-pore mesoporous silica were prepared and tested as adsorbents for the removal of representative inorganic (metal ions) and organic (dyes) water pollutants. Among the materials tested, MSNPs have proven to be a highly efficient and versatile adsorbent, exhibiting exceptional efficiency in the removal of both metal ions (Fe^3+^, Ni^2+^ and Cu^2+^) as well as organic dyes (MB and MG). The adsorption studies allowed to establish important relationships between surface charge and textural properties on the adsorptive performance of the mesoporous silica materials towards inorganic and organic pollutants. The kinetic studies with MSNPs and LPMS as adsorbents revealed that the dye adsorption process follows a pseudo-second-order kinetic model. The remarkable reusability of MSNPs was assessed through consecutive adsorption–desorption cycles and the exceptional stability of the adsorbent after use could also be confirmed. The present work provides relevant insights on the influence of a vast set of physicochemical properties of mesoporous silica materials on pollutant uptake, thereby allowing us to maximise their adsorptive performance for future practical applications in wastewater treatment and to reduce water pollution.

## Figures and Tables

**Table 1:** Elemental analysis data for the APTES- and TESPIC-functionalised silica materials. See Experimental Section for materials abbreviation meaning.

Material	Element Content (wt%)			Functional Group Content (mmol/g)
	C	H	N	
MSNPs-APTES	10.72	2.81	3.41	2.43
MSNPs-TESPIC	9.85	2.14	1.52	1.08
LPMS-APTES	6.10	1.57	1.90	1.35
LPMS-TESPIC	4.53	0.99	1.00	0.72

**Table 2:** Textural parameters of the mesoporous silica-based materials. See Experimental Section for materials’ abbreviation meanings.

Material	S_BET_ (m^2^/g)	V_p_ (cm^3^/g)	D_p_ (nm)
MSNPs	913	1.64	5.96
MSNPs-APTES	444	0.90	11.61
MSNPs-TESPIC	585	0.84	8.98
LPMS	138	0.75	16.36
LPMS-APTES	73	0.55	25.56
LPMS-TESPIC	109	0.64	19.99

S_BET_: specific surface area; V_p_: total pore volume determined at the relative pressure of 0.99; D_p_: pore diameter.

**Table 3:** Values of pH at the point of zero charge (pH_PZC_) of the mesoporous silica-based materials.

Material	pH_PZC_		
	As-Prepared	TESPIC	APTES
MSNPs	2.2	3.5	8.0
LPMS	2.1	4.9	9.2

**Table 4:** Kinetic parameters for the adsorption of methylene blue (MB) and methyl green (MG) organic dyes onto the MSNP and LPMS adsorbents.

Adsorbent	Dye	q_e,exp_ (mg g^−1^)	Pseudo-First-Order			Pseudo-Second-Order		
			q_e,cal_ (mg g^−1^)	k_1_ (min^−1^)	R^2^	q_e,cal_ (mg g^−1^)	k_2_ (g mg^−1^ min^−1^)	R^2^
MSNPs	MB	4.0656	0.0680	0.0138	0.651	3.9851	0.3253	0.999
MSNPs	MG	5.6316	0.6245	0.0356	0.849	5.4910	0.1969	0.999
LPMS	MB	3.3226	1.5473	0.0375	0.980	3.3914	0.0751	0.999
LPMS	MG	2.1128	1.3473	0.0198	0.985	2.1730	0.0410	0.992

**Table 5:** Comparison of the adsorption capacities (q_e_) of different silica-based adsorbents.

Adsorbent	Dye	q_e_ (mg/g)	t_e_ (min)	Reference
Silica nanosheets	MB	11.77	90	[65]
MCM-41	MB	55.0	300	[66]
SBA-15	MB	45.1	30	[67]
MCM-41	MB	11.83	240	[68]
MCM-48	MB	10.56	240	[68]
MCM-41	MG	20.97	1000	[69]
SBA-15-S_4_	MG	39.4	8	[70]
MSNPs	MB	4.07	20	This work
MSNPs	MG	5.63	20	This work

## Data Availability

Not applicable.

## Supplementary Materials

The following supporting information can be downloaded at: https://www.mdpi.com/article/10.3390/molecules28104038/s1, Figure S1: FT-IR spectra of (A) APTES-functionalized and (B) TESPIC-functionalized MSNP and LPMS mesoporous silica materials; Figure S2. Energy-dispersive X-ray spectroscopy spectra of (A) MSNP, (B) LPMS, (C) MSNP-APTES, (D) LPMS-APTES, (E) MSNP-TESPIC and (F) LPMS-TESPIC; Figure S3: Nitrogen adsorption-desorption isotherms at −196 ºC of (A) MSNP and (B) LPMS materials, before and after functionalization; Figure S4: Zeta potential values for (A) MSNP-type materials and (B) LPMS-type materials in the 2-12 pH range; Figure S5: Hydrodynamic sizes determined by DLS of the (A) MSNP-type and (B) LPMS-type materials in the 2-12 pH range; Figure S6. Precipitation percentage in blank experiments for (A) Ni2+, (B) Fe3+ and (C) Cu2+ over the 2-12 pH range; Figure S7: Removal efficiency for methylene blue (5 mg/L) by the mesoporous silica materials (0.5 g/L) with contact time of 2 h at room temperature; Figure S8: Effect of contact time on the adsorption of methylene blue (MB) and methyl green (MG) (5 mg/L) by MSNP and LPMS materials (1.0 g/L); Figure S9: (A) Pseudo-first-order and (B) Pseudo-second-order kinetics model for the adsorption of organic dyes (methylene blue MB and methyl green MG) using MSNP and LPMS materials; Figure S10: FT-IR spectra of the as-prepared MSNP material and after adsorption studies with methylene blue; Figure S11: SEM micrographs of MSNP material after the first cycle with methylene blue (A) and methyl green (B), and after the third adsorption cycle with methylene blue (C) and methyl green (D); Table S1: Assignment of the vibrational FT-IR bands of the mesoporous silica materials.
