# Supplementary material for: Tailoring of Mesoporous Silica-Based Materials for Enhanced Water Pollutants Removal
Source: Molecules. 2023 May 11;28(10):4038. doi: 10.3390/molecules28104038 (PMC10223029; doi:10.3390/molecules28104038)
Supplement: Supplementary file 1 [file molecules-28-04038-s001.zip › molecules-2330138-supplementary.pdf]

ELECTRONIC SUPPORTING INFORMATION

Tailoring of Mesoporous Silica-Based Materials for Enhanced Water Pollutants Removal

Daniela Flores,[1] C. Marisa R. Almeida,[2] Carlos Rocha Gomes,[2] Salete S. Balula,[1] Carlos M. Granadeiro[1,*]

[1]LAQV-REQUIMTE, Departamento de Química e Bioquímica, Faculdade de Ciências, Universidade do Porto, Rua do Campo Alegre, s/n 4169-007 Porto, Portugal

[2]Centro Interdisciplinar de Investigação Marinha e Ambiental (CIIMAR), Universidade do Porto, Terminal de Cruzeiros do Porto de Leixões, Av. General Norton de Matos s/n, 4450-208 Matosinhos, Portugal and Departamento de Química e Bioquímica, Faculdade de Ciências, Universidade do Porto, Rua do Campo Alegre, 687, 4169-007 Porto, Portugal

Table S1. Assignment of the vibrational FT-IR bands of the mesoporous silica materials.

Material	v_s(C-H)	δ(N-H)	v_{as}(Si-O-Si)	v_s(Si-O-Si)	δ(O-Si-O)
MSNP	-	-	1078	794	456
MSNP-APTES	2929	1558	1074	795	461
MSNP-TESPIC	2980; 2931	1574	1074	800	455
LPMS	-	-	1076	805	456
LPMS-APTES	2958	1558	1105	808	471
LPMS-TESPIC	2989	1541	1103	808	471

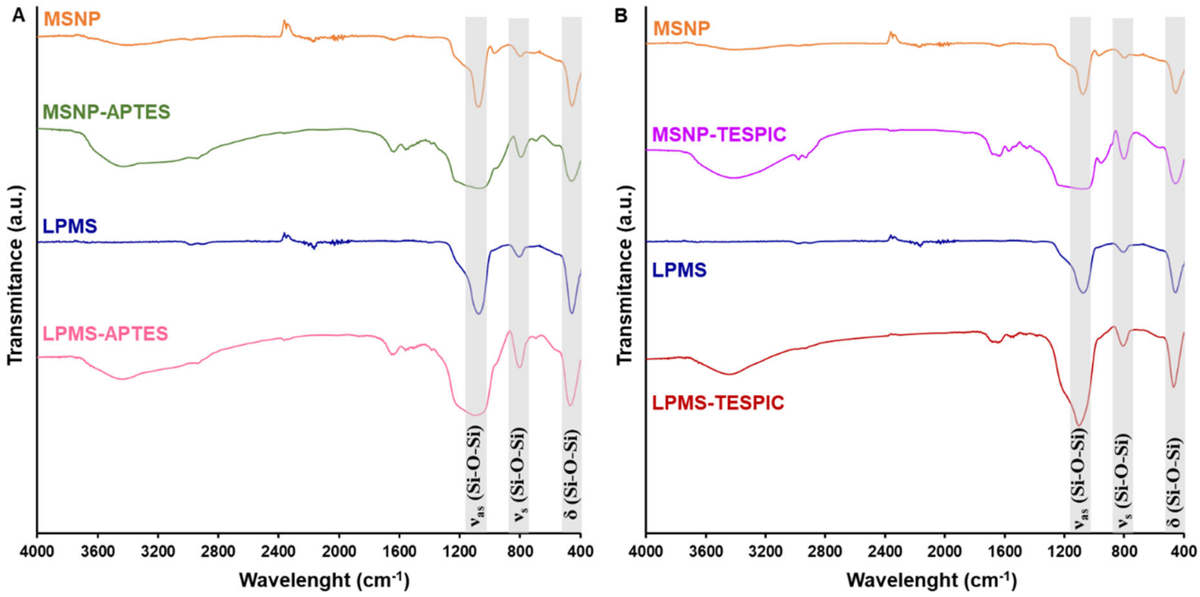

Figure S1. FT-IR spectra of (A) APTES-functionalized and (B) TESPIC-functionalized MSNP and LPMS mesoporous silica materials. See the Experimental section for materials abbreviation meaning.

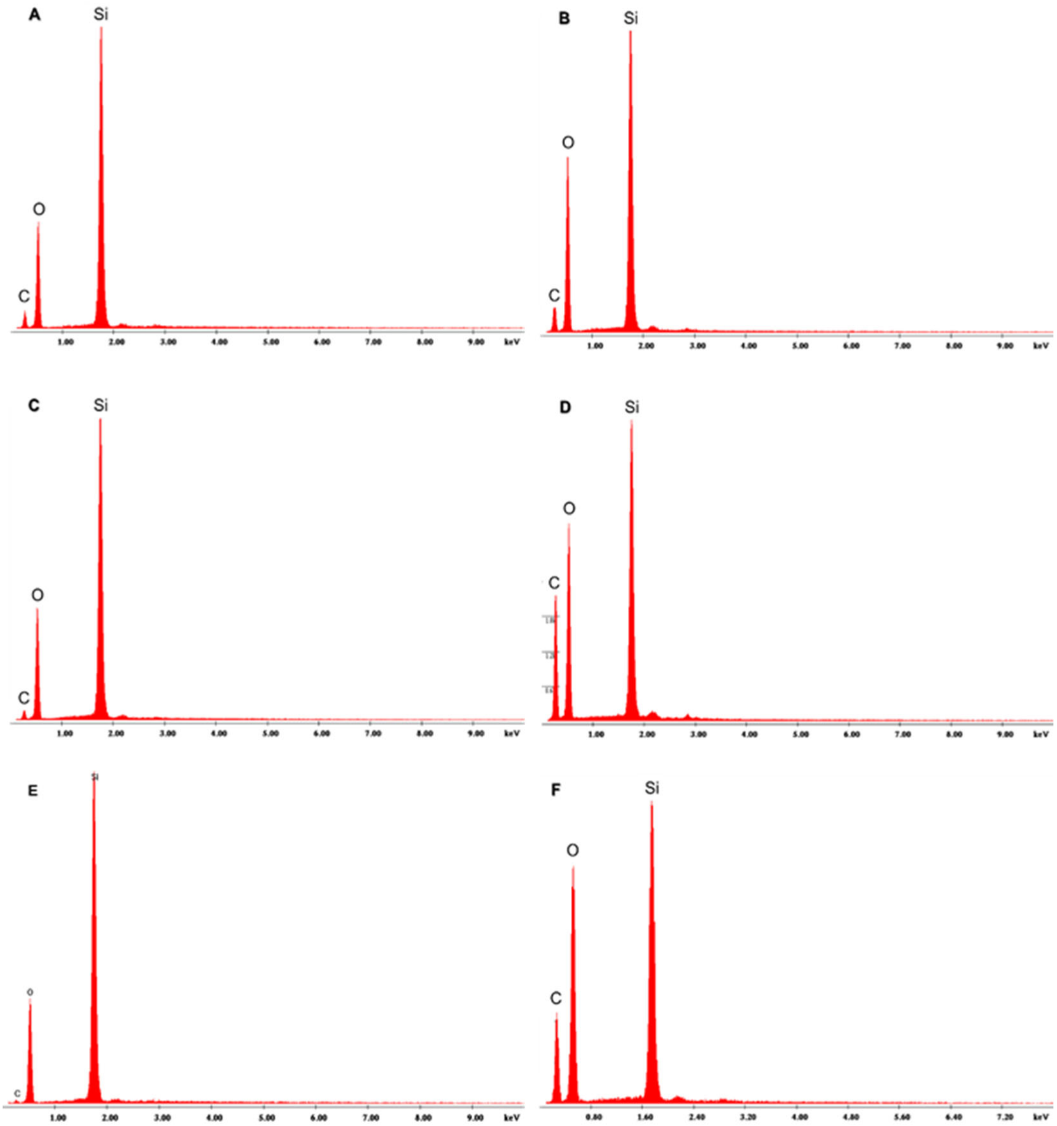

Figure S2. Energy-dispersive X-ray spectroscopy spectra of (A) MSNP, (B) LPMS, (C) MSNP-APTES, (D) LPMS-APTES, (E) MSNP-TESPIC and (F) LPMS-TESPIC. See the Experimental section for materials abbreviation meaning.

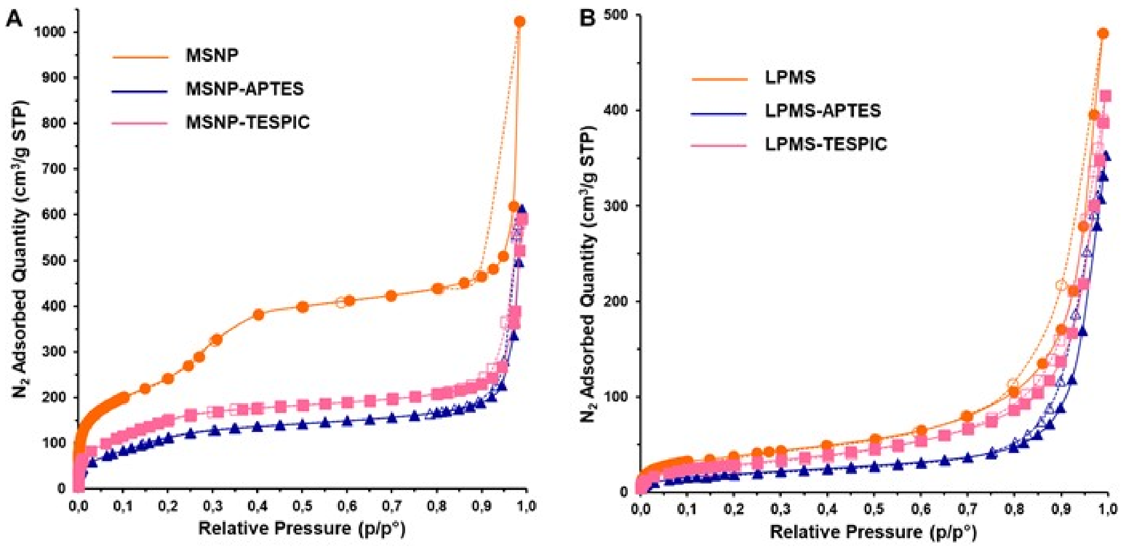

Figure S3. Nitrogen adsorption-desorption isotherms at -196 ºC of (A) MSNP and (B) LPMS materials, before and after functionalization. Filled and unfilled symbols represent the adsorption and desorption of nitrogen, respectively. See the Experimental section for materials abbreviation meaning.

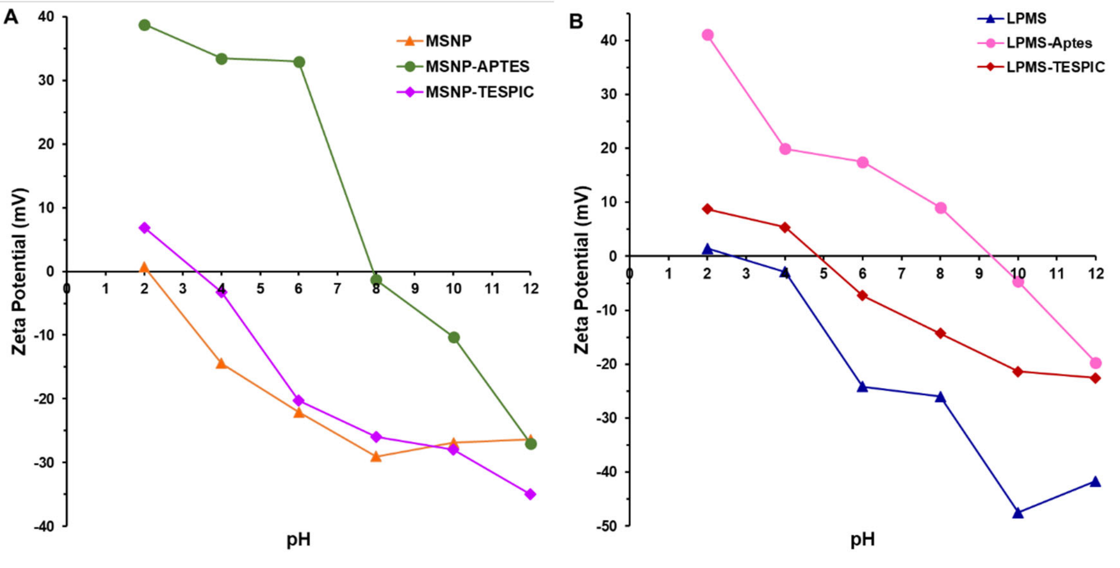

Figure S4. Zeta potential values for (A) MSNP-type materials and (B) LPMS-type materials in the 2-12 pH range. See the Experimental section for materials abbreviation meaning.

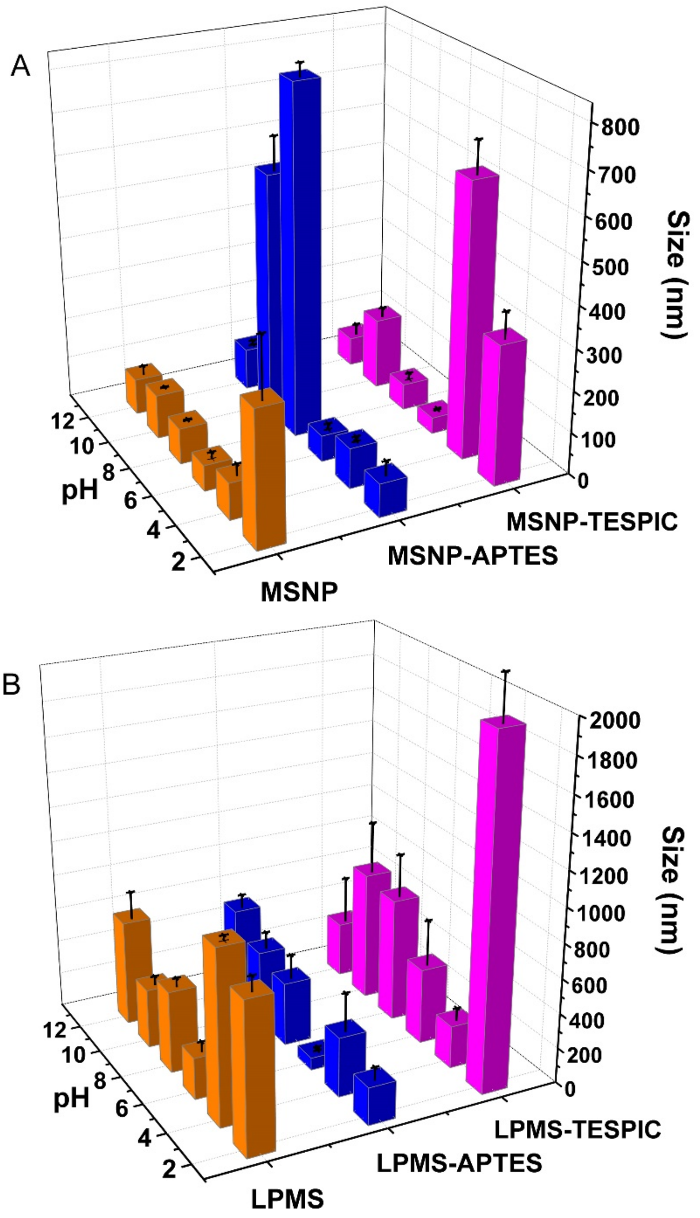

Figure S5. Hydrodynamic sizes determined by DLS of the (A) MSNP-type and (B) LPMS-type materials in the 2-12 pH range. See the Experimental section for materials abbreviation meaning.

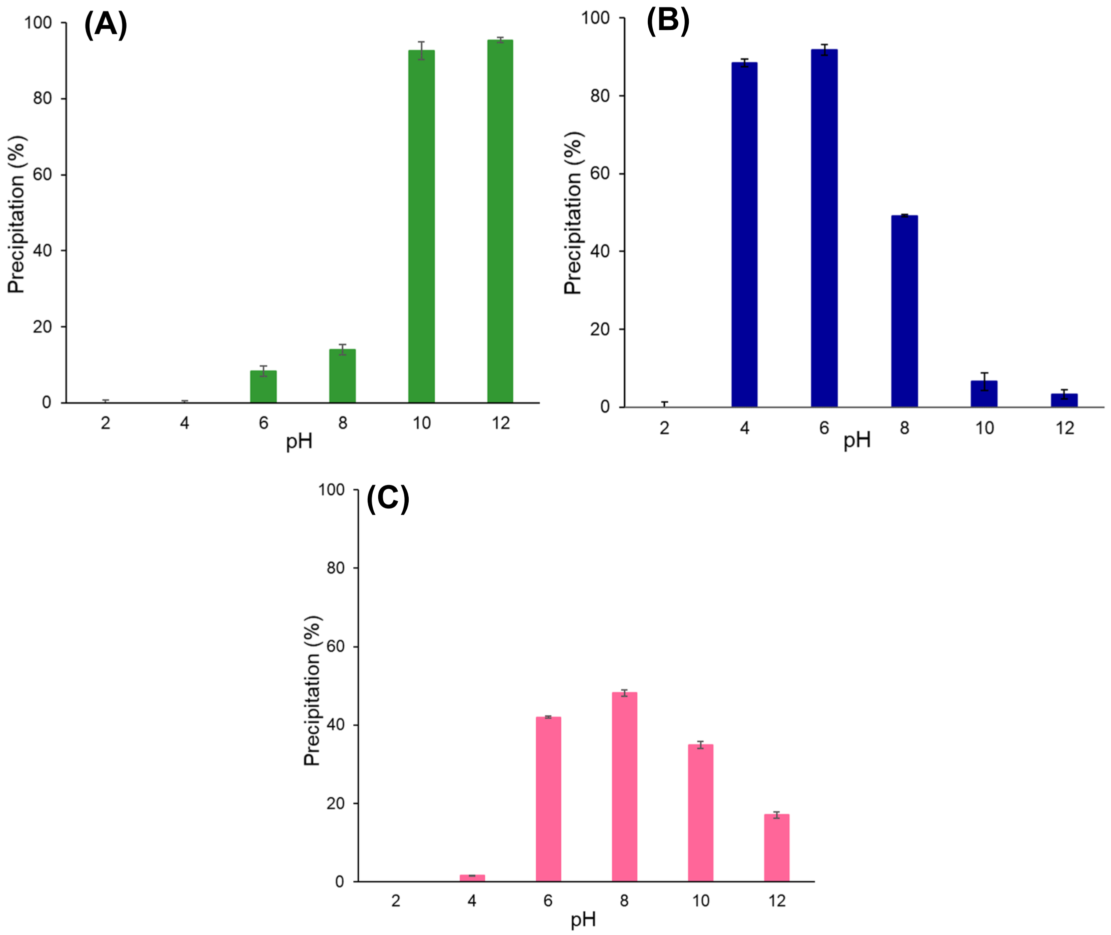

Figure S6. Precipitation percentage in blank experiments for (A) Ni^{2+}, (B) Fe^{3+} and (C) Cu^{2+} over the 2-12 pH range. Mean and standard (n=3) are shown.

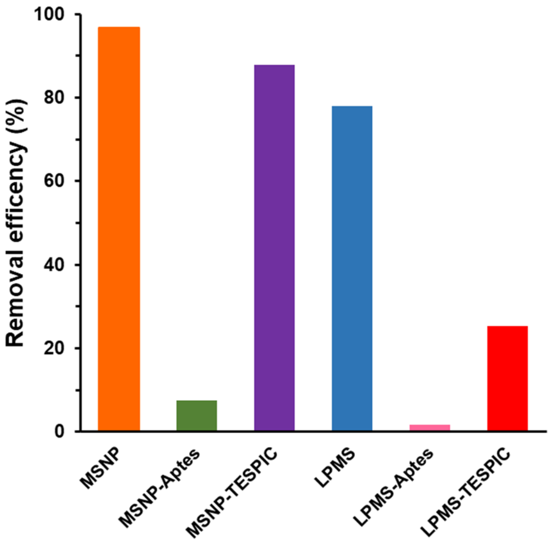

Figure S7. Removal efficiency for methylene blue (5 mg/L) by the mesoporous silica materials (0.5 g/L) with contact time of 2 h at room temperature. Mean and standard (n=3) are shown. See the Experimental section for materials abbreviation meaning.

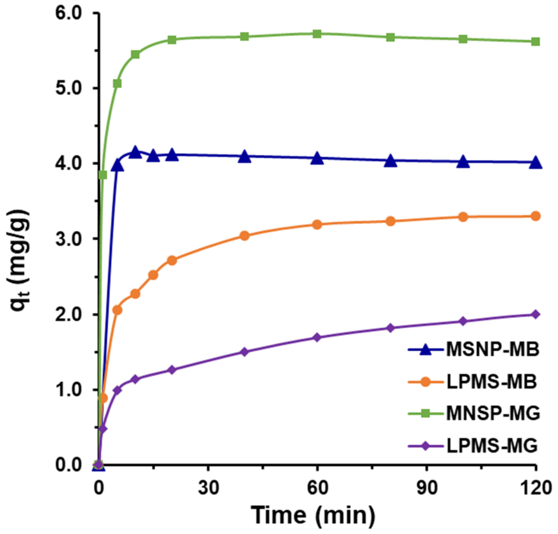

Figure S8. Effect of contact time on the adsorption of methylene blue (MB) and methyl green (MG) (5 mg/L) by MSNP and LPMS materials (1.0 g/L). See the Experimental section for materials abbreviation meaning.

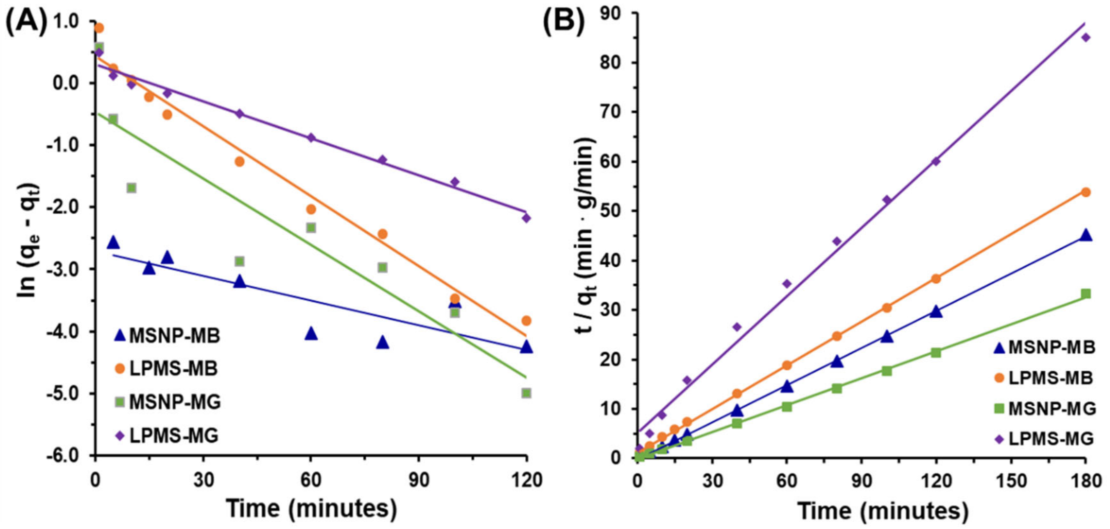

Figure S9. (A) Pseudo-first-order and (B) Pseudo-second-order kinetics model for the adsorption of organic dyes (methylene blue MB and methyl green MG) using MSNP and LPMS materials. See the Experimental section for materials abbreviation meaning.

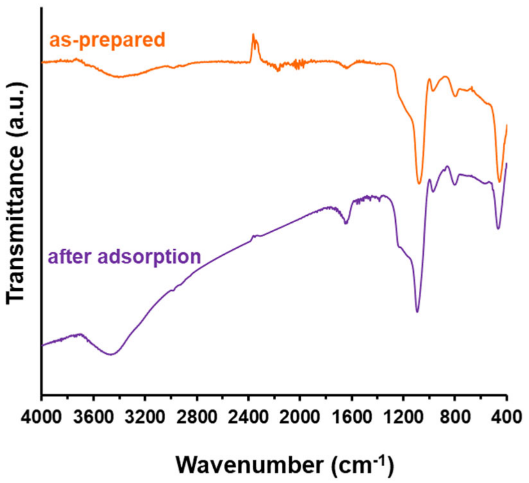

Figure S10. FT-IR spectra of the as-prepared MSNP material and after adsorption studies with methylene blue.

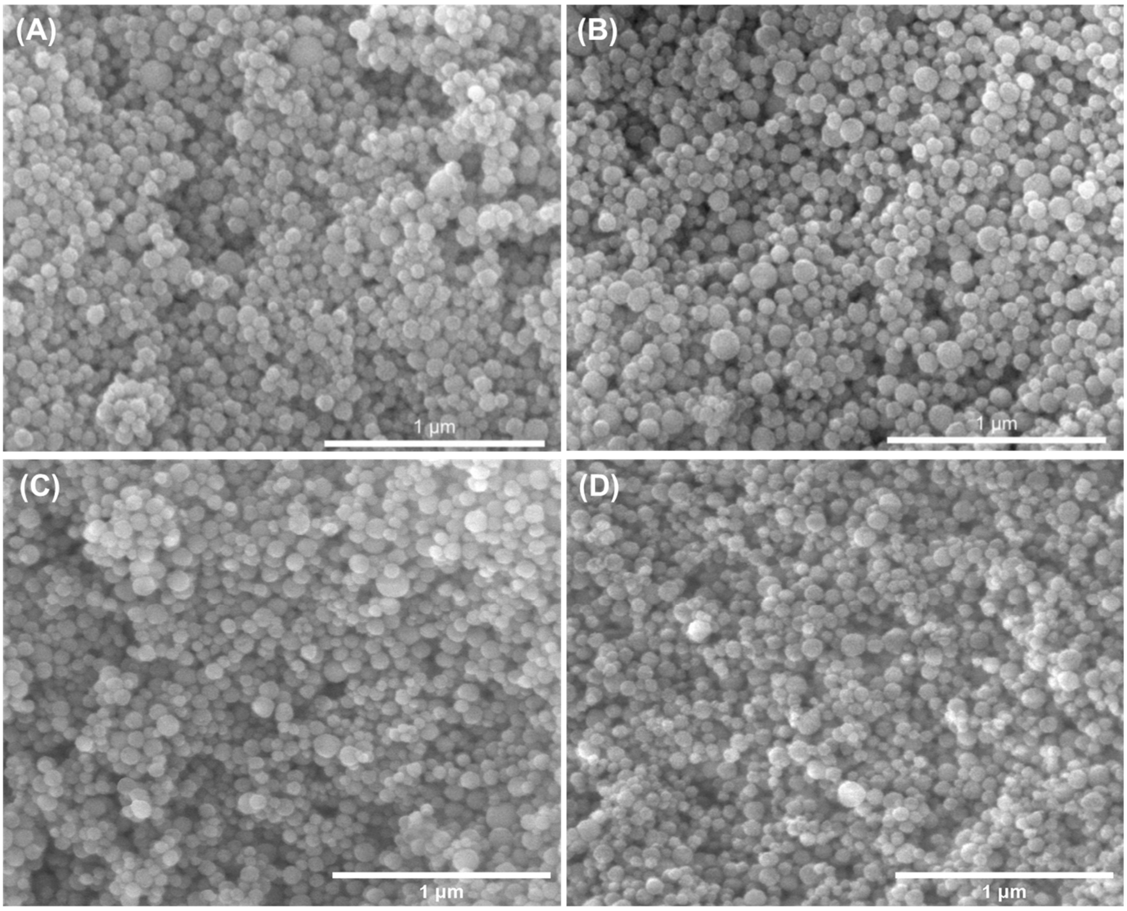

Figure S11. SEM micrographs of MSNP material after the first cycle with methylene blue (A) and methyl green (B), and after the third adsorption cycle with methylene blue (C) and methyl green (D).
